# Gas embolism in double-balloon endoscopic retrograde cholangiography with carbon dioxide insufflation

**DOI:** 10.1055/a-2446-1986

**Published:** 2024-11-11

**Authors:** Yuki Kawasaki, Susumu Hijioka, Yoshikuni Nagashio, Mark Chatto, Takuji Okusaka, Yutaka Saito

**Affiliations:** 1 68380 Department of Hepatobiliary and Pancreatic Oncology, National Cancer Center Hospital, Chuo-ku, Japan; 2 378609 Digestive Disease Center, Showa University Koto Toyosu Hospital, Koto-ku, Japan; 3 37571 Department of Gastroenterology, Makati Medical Center, Makati City, Philippines; 4 68380 Endoscopy Division, National Cancer Center Hospital, Chuo-ku, Japan

Gas embolism during endoscopy is a rare but potentially fatal adverse event. Some risk factors, such as biliary procedures and gastrointestinal reconstruction have been cited 1 . The use of carbon dioxide (CO _2_ ) insufflation is effective in preventing gas embolism, although a few cases of gas embolism with CO _2_ insufflation during balloon-assisted endoscopic retrograde cholangiography (ERC) have been reported 2 3 .

A 70-year-old male patient had undergone pancreaticoduodenectomy for a pancreatic neuroendocrine tumor (pNET) 11 years previously. The pNET showed recurrent liver metastases 3 years after surgery, and chemotherapy was initiated. Two biliary plastic stents were placed in the posterior branch during balloon-assisted ERC for malignant bile duct strictures 10 years after surgery. At 1 year later, the patient was admitted to the hospital with biloma in the anterior hepatic segment ( Fig. 1 **a, b** ).

**Fig. 1:**
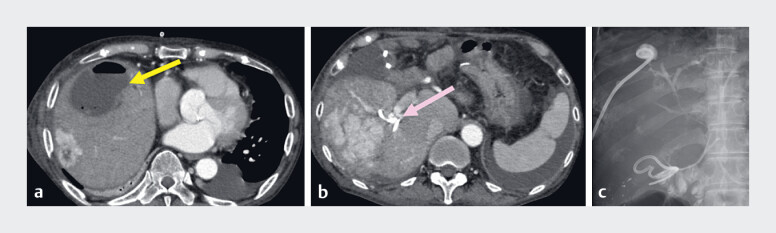
**a** Computed tomography (CT) on admission showed an abscess with air in the anterior hepatic segment (yellow arrow); and **b** two remaining biliary plastic stents in the posterior bile duct (pink arrow). **c** Contrast-enhanced imaging, with medium introduced via the percutaneous drainage tube, showed communication with the bile duct.

Contrast-enhanced imaging, with contrast introduced via the drainage tube for the biloma, showed communication with the bile duct ( Fig. 1 **c** ). We therefore attempted endoscopic management with double-balloon enteroscopy (DBE) (EI-580BT; Fujifilm, Japan) ( Video 1 ). During the removal of the remaining stents ( Fig. 2 **a, b** ), the patient suddenly went into shock. Spontaneous respiration stopped and cardiopulmonary resuscitation was initiated. Spontaneous respiration recovered 10 minutes later, and computed tomography (CT) showed gas in the right atrium and in a tumor in the posterior hepatic segment ( Fig. 3 **a, b** ). CT of the head revealed no obvious gas embolism ( Fig. 3 **c** ). On CT done 2 h after the procedure, the intracardiac gas had disappeared, and the intratumoral gas had also decreased ( Fig. 4 **a, b** ).

**Fig. 2:**
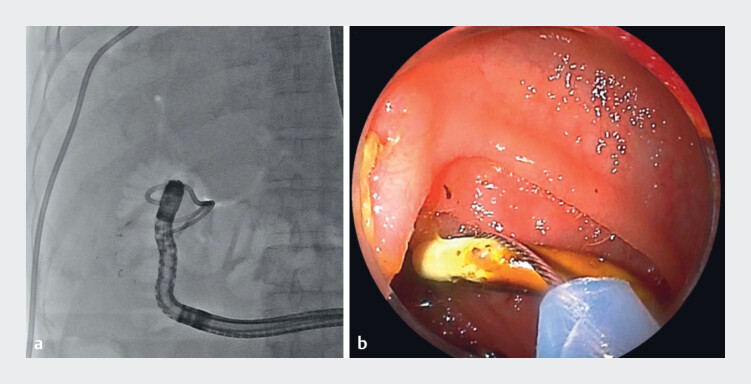
Removal of plastic biliary stents previously placed for malignant duct strictures. **a** Fluoroscopic image. **b** Endoscopic view.

**Fig. 3:**
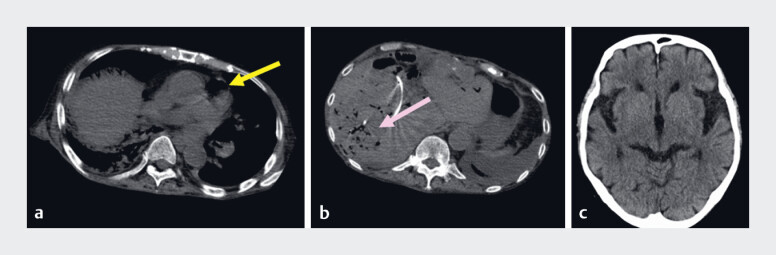
**a** CT immediately after resuscitation showed gas in the right atrium (yellow arrow), and **b** in a tumor in the posterior hepatic segment (pink arrow). **c** Head CT showed no obvious gas embolism.

**Fig. 4:**
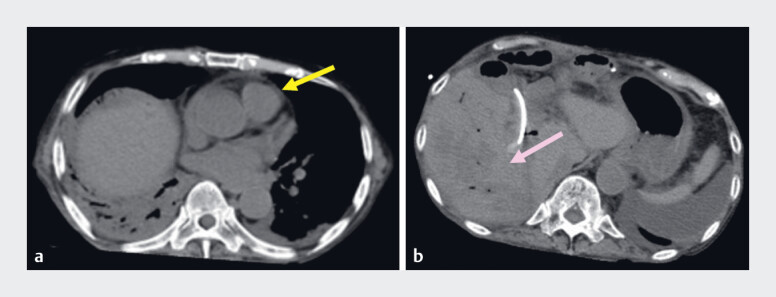
a Computed tomography done 2 h after resuscitation showed: **a** disappearance of gas in the right atrium (yellow arrow), and **b** decrease of gas in the tumor in the posterior hepatic segment (pink arrow).


Gas embolism via hepatic hypervascular tumor, with carbon dioxide (CO _2_ ) insufflation for double-balloon enteroscopy.
Video 1

CO _2_ influx from the DBE insufflation, passing from the bile duct via the hypervascular tumor into the portal vein ( Fig. 5 ), was believed to be the cause of the gas embolism, as suggested by the imaging findings. Patients with hepatic hypervascular tumors, as well as those with reconstructed bowels, have a high risk for gas embolism, even with CO _2_ insufflation.

**Fig. 5:**
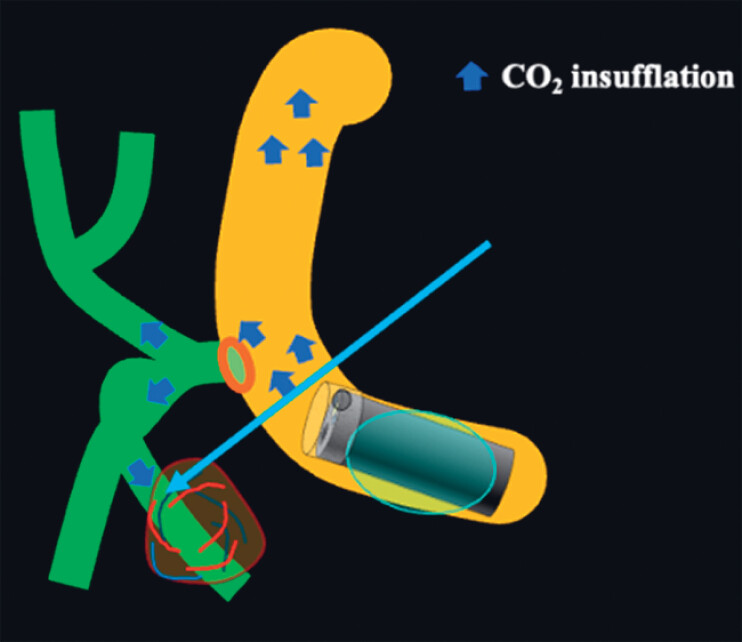
Schema showing carbon dioxide (CO _2_ ; arrowheads) from the double-balloon enteroscopy insufflation, passing from the bile duct through the hypervascular tumor and into the portal vein (arrow).

Endoscopy_UCTN_Code_CPL_1AK_2AC

## Notes

Endoscopy E-Videos https://eref.thieme.de/e-videos
*E-Videos* is an open access online section of the journal *Endoscopy* , reporting on interesting cases and new techniques in gastroenterological endoscopy. All papers include a high-quality video and are published with a Creative Commons CC-BY license. Endoscopy E-Videos qualify for HINARI discounts and waivers and eligibility is automatically checked during the submission process. We grant 100% waivers to articles whose corresponding authors are based in Group A countries and 50% waivers to those who are based in Group B countries as classified by Research4Life (see: https://www.research4life.org/access/eligibility/ ). This section has its own submission website at https://mc.manuscriptcentral.com/e-videos .
